# Supplementary material for: Topic and Sentiment Trends in Semaglutide Discussions on X: Subpopulation-Based Longitudinal Analysis
Source: Online J Public Health Inform. 2026 Feb 24;18:e80660. doi: 10.2196/80660 (PMC12976598; doi:10.2196/80660)
Supplement: Multimedia Appendix 1 [file ojphi_v18i1e80660_app1.docx]

#### **User Subpopulation Tables**

**Table S1** summarizes the user subpopulations and lists the number of posts and number of users belonging to each subpopulation. Users are classified into subpopulations based on their demographic attributes, which are inferred by Brandwatch. The Unknown category accounts for cases where no inference could be made or the data is missing or null.

**Table S1.** Summary of the user subpopulations, along with the size of each subpopulation (measured via number of posts and number of users).

| Attribute | Subpopulation | Number of Posts | Number of Users |
| --- | --- | --- | --- |
|  |  |  |  |
| Gender | Male | 202,042 | 105,591 |
|  | Female | 170,997 | 87,556 |
|  | Unknown | 486,712 | 243,404 |
|  |  |  |  |
| Verified | True | 78,649 | 27,410 |
|  | False | 733,361 | 393,532 |
|  | Unknown | 47,741 | 15,609 |
|  |  |  |  |
| Account Type | Individual | 816,227 | 426,691 |
|  | Organizational | 43,524 | 9,860 |
|  | Unknown | 0 | 0 |
|  |  |  |  |
| US Region | Midwest | 33,974 | 17,491 |
|  | Northeast | 72.505 | 30,840 |
|  | Southeast | 49.403 | 25,260 |
|  | Southwest | 30.024 | 15,775 |
|  | West | 52.790 | 25,482 |
|  | Unknown | 0 | 0 |

Table S2 presents the user interest subpopulations. To divide users by interest, we begin by parsing the interest column in the dataset, which contains comma-separated interest groups. For each user, we aggregate all interests that are applied to any of their posts. A single user may therefore contribute to multiple interest categories. In the per-post analysis, we count the number of times each interest appears across posts.

**Table S2**. Number of posts and users per interest.

| Interest | Number of Posts | Number of Users |
| --- | --- | --- |
| Family & Parenting | 64,440 | 32,136 |
| Beauty/Health & Fitness | 61,524 | 19,979 |
| Politics | 53,751 | 25,932 |
| Books | 50,167 | 21,327 |
| Business | 48,188 | 20,649 |
| Sports | 42,939 | 21,739 |
| Animals & Pets | 28,469 | 13,250 |
| Music | 28,043 | 14,993 |
| Science | 26,868 | 10,682 |
| Technology | 24,607 | 11,588 |
| Food & Drinks | 22,798 | 11,220 |
| Fine arts | 15,531 | 8,251 |
| TV | 15,449 | 6,394 |
| Movies | 10,699 | 5,315 |
| Games | 10,624 | 6,206 |
| Environment | 8,134 | 3,733 |
| Travel | 8,004 | 3,897 |
| Photo & Video | 7,878 | 3,763 |
| Automotive | 4,932 | 2,639 |
| Fashion | 4,656 | 2,103 |
| Shopping | 3,538 | 1,499 |

#### **Dataset Availability**

An anonymized version of our dataset, which has the “Author”, “Avatar”, “Entity Info”, “Url”, “Original Url”, “Full Name”, “Twitter Retweet of”, “Twitter Reply to”, “Thread Author”, and “Weblog Title” columns removed, may be downloaded using the following link:

<https://www.dropbox.com/scl/fi/gksibelrijwnr1wg6xja2/dataset_anonymized.csv?rlkey=ks6dj60ysan9lwh8wdqnygma4&st=294q0sib&dl=0>.

#### **Elbow Method Figures**

**Figure S1**. K-means clustering results for the positive document corpus.


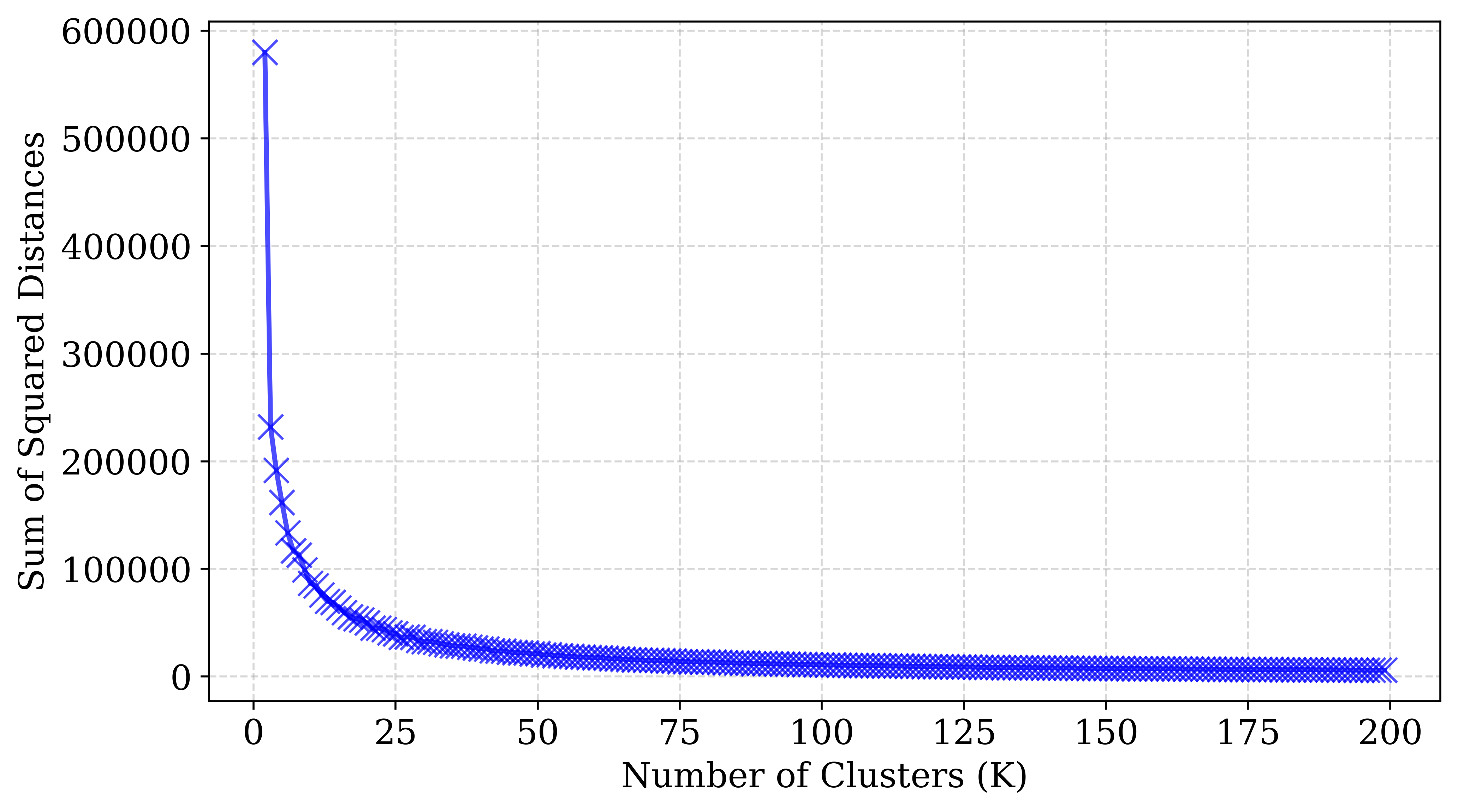


**Figure S2.** K-means clustering results for the negative document corpus.


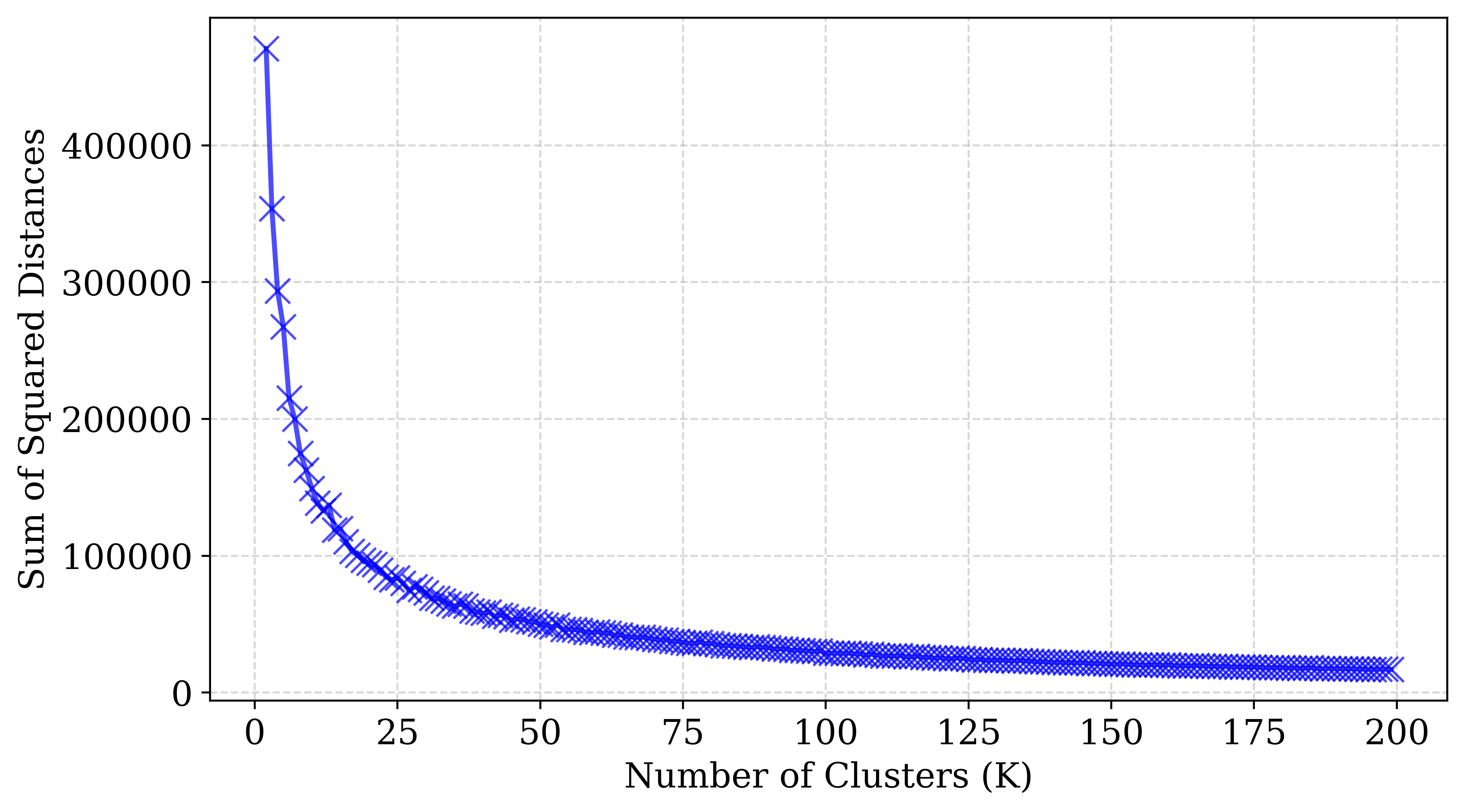


#### **Topic Dictionary**

Multimedia Appendix 2 provides the mapping of the 200 BERTopic clusters to the ten umbrella topics. For each of the 200 initial topics, this file displays the number of posts associated with the topic, representative keywords and posts, and the umbrella topic mappings chosen by the annotators. The topics are sorted by post count, and the 100 positive topics appear before the 100 negative topics.

In addition, Table S3 provides example representative documents (posts) for each of the ten umbrella topics. Note that the provided representative documents are not original posts, but posts that have underwent the cleaning steps discussed in the BERTopic Modeling section.

**Table S3**. Number, Name, and representative documents for each of the ten umbrella topics.

| Topic Number | Topic Name | Example Representative Documents |
| --- | --- | --- |
| T0 | Weight Loss | ['ready lose weight gain confidence say hello semaglutide gamechanging prescription medication help achieve significant weight loss with semaglutide take control craving appetite finally reach weight loss goal', 'semaglutide weight loss', 'have hear new semaglutide weight loss injection semaglutide onceweekly injection provide amazing weight loss result to learn semaglutide check website'] |
| T1 | Celebrities/politicians | ['ccrook oprah oprah ozempic weight loss', 'o o o ozempic can not stop sing o o o oprah special really think differently weight loss possible thank oprah', 'oprah you go girl im think gain weight back i take ozempic eat gummies become transform like oprah and i want pantsuit like oprah oprah'] |
| T2 | Obtaining the drug | ['afrosabi have prescription ozempic we help help thousand americans save up to insulin drug save first order coupon code first', 'americans save up to insulin drug ozempic save first order coupon code first', 'americans save up to insulin medication ozempic save first order coupon code first'] |
| T3 | Drug indicators | ['ozempic wegovy may help treat alcohol addiction researcher say phillyvoice weightloss drug may helpful treat people alcohol addiction addictive disorder accord new research medication', 'could ozempic help people addiction alcohol nicotine one study today say actually might', 'could ozempic cure other addictive behaviors glp drug like ozempic effective treat food addiction recent report suggest drug may helpful addiction this may especially true reduce alcohol intake alcohol use disorder'] |
| T4 | Drug authorities | ['novo nordisk see fast profit growth demand diabetes drug ozempic', 'ozempic get even good novo nordisk stock', 'novo nordisk danish maker weightloss drug wegovy become valuable company europe'] |
| T5 | General and profane  negativity | ['i dont give shit ozempic', 'oh oh oh ozempic say', 'damn you ozempic'] |
| T6 | Death | ['the eu look report ozempic drug cause suicidal thought', 'eu investigate ozempic weightloss drug saxenda suicidal thought report pharma suicide medicine obesity', 'wegovy ozempic weightloss drug cause suicidal thought'] |
| T7 | Chronic harm | ['show cause thyroid tumor thyroid cancer animal it know ozempic cause thyroid tumor medullary thyroid cancer human', 'serious side effect ozempic include thyroid tumor pancreatitis change vision hypoglycemia gallbladder issue kidney failure thyroid cancer in animal study ozempic increase risk thyroid tumor', 'ozempic thyroid cancer'] |
| T8 | Acute harm/adverse  drug reactions | ['does ozempic mental health side effect many people take ozempic related glp weight loss drug describe mentalhealth problem but side effect isnt mention ozempics instruction use drug label are problem coincidence relate', 'all drug come risk side effect', 'a lot people talk ozempic moment i use month cause terrible side effect i use month still suffer side effect there quick fix the pharma industry friend'] |
| T9 | Other | ['is catchy song time ozempic jingle', 'i glad bring back old school commercial great commercial song', 'ozempic definitely favorite prescription drug commercial song', 'this important article', 'check article', 'interesting article', 'happy thanksgiving this message bring ozempic we expect eat less', 'ozempic enjoy two bite thanksgiving dinner me', 'ozempic thanksgiving yes'] |

***Umbrella Topic Stability Checks***

To verify that the umbrella topic shares do not change significantly under different cluster counts, we run the BERTopic model on our document corpora using cluster counts of 25 and 50, obtaining topic and document representations. These stability checks are run under varying random seeds to ensure that the seed does not significantly affect the results. We repeat the manual umbrella topic annotation exercise (described in the BERTopic Modeling section) for these cluster counts. The intercoder agreements for the manual annotation of the 25 and 50 clusters are 0.808 and 0.822, respectively. Table S4 shows the umbrella topic shares (i.e., the number of posts mapped to each of the ten umbrella topics) under our cluster counts of 25, 50, and 100.

**Table S4**. Umbrella topic shares for cluster counts of 25, 50, and 100.

| Topic Number | Number of Posts  (25 clusters) | Number of Posts  (50 clusters) | Number of Posts  (100 clusters) |
| --- | --- | --- | --- |
| T0 | 55213 | 54087 | 50542 |
| T1 | 38671 | 40152 | 40120 |
| T2 | 19423 | 24811 | 22724 |
| T3 | 18477 | 13207 | 16948 |
| T4 | 18398 | 20863 | 23783 |
| T5 | 8830 | 12672 | 9488 |
| T6 | 1168 | 2357 | 1486 |
| T7 | 2987 | 2568 | 3075 |
| T8 | 14946 | 10660 | 10208 |
| T9 | 29928 | 26664 | 29475 |

#### **Sensitivity Analysis — Detailed Results**

To assess the robustness of our findings, we conducted a sensitivity analysis excluding reposts from the dataset, which reduced the total sample to 411,747 tweets. The results confirmed that demographic, verification, and regional sentiment patterns were not artifacts of repost amplification. Gender-based differences remained consistent, with female users expressing more negative attitudes than male users across both analyses. Verified and organizational accounts continued to exhibit less negative sentiment compared to non-verified and individual users, respectively. Regional variation also persisted, with the Southeast, Midwest, and Southwest showing the most negativity, while the Northeast and West expressed comparatively less negative sentiment. Overall, these results demonstrate that excluding reposts did not alter the primary conclusions, reinforcing the robustness of our findings. Table S5 summarizes these results, showing consistent subgroup sentiment patterns across analyses with and without reposts.

**Table S5**. Sentiment patterns across user subpopulations, comparing analyses with and without reposts. Female users expressed more negative sentiment in both analyses, verified users were less negative, and organizations remained near neutral.

| Subpopulation | With Reposts | Without Reposts |
| --- | --- | --- |
| Gender |  |  |
|  | Female (−0.292 post; −0.323 user) | Female (−0.232 post; −0.263 user) |
|  | Male (−0.167 post; −0.200 user) | Male (−0.151 post; −0.181 user) |
|  | Unknown (−0.256 post; −0.300 user) | Unknown (−0.186 post; −0.247 user) |
| Verified |  |  |
|  | Verified (−0.143 post; −0.174 user) | Verified (−0.121 post; −0.157 user) |
|  | Nonverified (−0.240 post; −0.282 user) | Nonverified (−0.177 post; −0.23 user) |
|  | Unknown (−0. 234 post; −0.282 user) | Unknown (−0.196 post; −0.253 user) |
| Account Type |  |  |
|  | Organizations(−0.014 post; −0.037 user) | Organizations(−0.009 post; −0.013 user) |
|  | Individuals(−0.242 post; −0.281 user) | Individuals (−0.187 post; −0.234 user) |
| US Region |  |  |
|  | Southeast (−0.261 post; −0.288 user) | Southeast (−0.189 post; −0.216 user) |
|  | Midwest (−0.250 post; −0.282 user) | Midwest (−0.199 post; −0.226 user) |
|  | Southwest (−0.254 post; −0.283 user) | Southwest (−0.185 post; −0.230 user) |
|  | West (−0.219 post; −0.253 user) | West (−0.164 post; −0.196 user) |
|  | Northeast (−0.195 post; −0.245 user) | Northeast (−0.155 post; −0.195 user) |

The overall temporal patterns also remained stable after excluding reposts. The general trends of sentiment declines and subsequent recoveries were preserved, indicating that repost exclusion did not alter the temporal dynamics of public discourse. These results are illustrated in Figure S3.

**Figure S3.** Bimonthly time-series plots of sentiment analysis categorized by gender, interest, verification status, account type, and region, with retweets excluded. To enhance readability, the interest visualization is limited to the top five most popular categories Shaded areas represent 95% confidence bands.


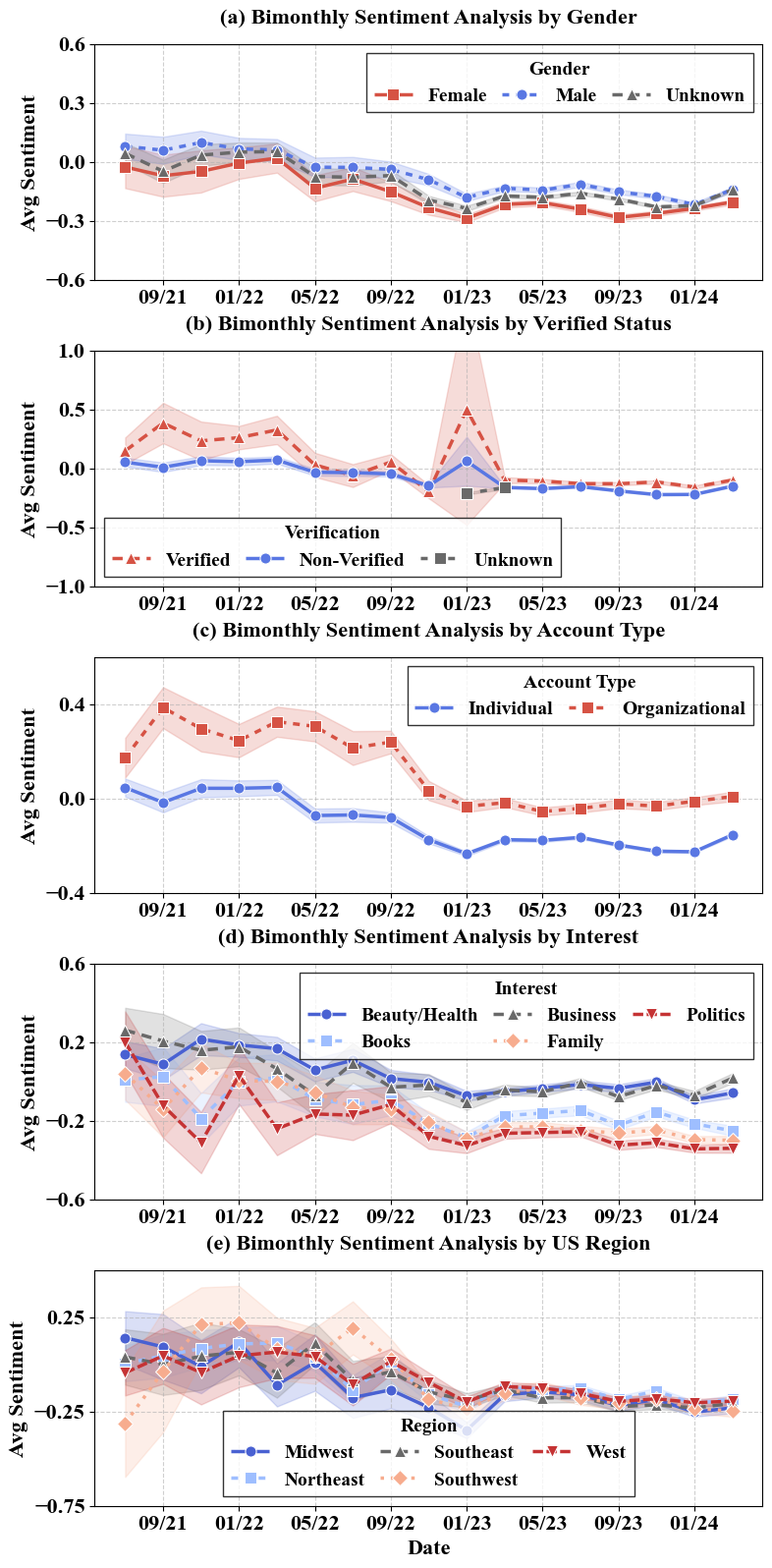


#### **Topic Modeling Detailed Results**

**Table S6**. ITS regression of T6 prevalence. The intervention point is set at July2023--September2023. PostTime indicates the slope change thereafter.

|  | β Coefficient | Standard Error | *P*-value | 95% CI |
| --- | --- | --- | --- | --- |
| Constant | -3.121 | 58.768 | 0.958 | [-130.081, 123.839] |
| Time | 0.801 | 7.985 | 0.922 | [-16.450, 18.051] |
| Event | 449.512 | 94.468 | < 0.001 | [245.426, 653.598] |
| PostTime | -83.1007 | 31.233 | 0.020 | [-150.576, -15.625] |

**Table S7**. Effect sizes and CIs for key topic prevalence contrasts.

| Topic | Prevalence | 95% CI | Effect Size |
| --- | --- | --- | --- |
| Male T1 | 0.175 | [0.172, 0.179] | 0.535 |
| Female T1 | 0.21 | [0.206, 0.214] | 0.538 |
| Male T4 | 0.134 | [0.131, 0.138] | 0.535 |
| Female T4 | 0.091 | [0.088, 0.094] | 0.538 |
| Verified T5 | 0.032 | [0.029, 0.034] | 0.535 |
| Nonverified T5 | 0.049 | [0.048, 0.050] | 0.534 |
| Verified T6 | 0.015 | [0.013, 0.016] | 0.535 |
| Nonverified T6 | 0.007 | [0.006, 0.007] | 0.534 |
| Verified T4 | 0.192 | [0.187, 0.197] | 0.535 |
| Nonverified T4 | 0.103 | [0.101, 0.104] | 0.534 |
| Organizational T5 | 0.008 | [0.007, 0.009] | 0.626 |
| Individual T5 | 0.049 | [0.048, 0.049] | 0.534 |
| Organizational T6 | 0.023 | [0.021, 0.026] | 0.626 |
| Individual T6 | 0.006 | [0.006, 0.006] | 0.534 |
| Organizational T4 | 0.257 | [0.249, 0.264] | 0.626 |
| Individual T4 | 0.104 | [0.102, 0.105] | 0.534 |
| Business T0 | 0.258 | [0.251, 0.266] | 0.602 |
| Business T4 | 0.268 | [0.261, 0.276] | 0.602 |
| Beauty/Health & Fitness T0 | 0.412 | [0.405, 0.420] | 0.642 |
